# Laparoscopic tumorectomy for a primary ovarian leiomyoma during pregnancy: A case report

**DOI:** 10.3892/ol.2014.2596

**Published:** 2014-10-09

**Authors:** XIAOFENG ZHAO, LIFENG CHEN, WENJIE ZENG, BIHUI JIN, WEIJIE DU

**Affiliations:** Department of Gynecology, Zhejiang Provincial People’s Hospital, Hangzhou, Zhejiang 310014, P.R. China

**Keywords:** laparoscopy, ovarian leiomyoma, pregnancy

## Abstract

Few reports have explored laparoscopic adnexal tumorectomy as a treatment for large and symptomatic ovarian leiomyomas during pregnancy. The current study presents the case of a patient with a large and symptomatic ovarian leiomyoma at 14 weeks of pregnancy. A laparoscopic adnexal tumorectomy was performed without complications. The laparoscopic management of large primary ovarian leiomyoma during pregnancy has not been reported in literature. Therefore, laparoscopy may be considered as a minimally invasive and feasible alternative to laparotomy for the treatment of large ovarian solid tumors during pregnancy, resulting in reduced postoperative pain, a smaller scar and shorter recovery time. By contrast, with respect to the ovarian solid tumor, surgery prior to gestation is advised, even for tumors of <3 cm in diameter, due to the probability of rapid growth of the tumor during pregnancy.

## Introduction

Leiomyoma is a rare ovarian tumors, which accounts for 0.5–1% of all benign ovarian tumors (1). They are usually bilateral if they occur in young patients, however, no bilateral ovarian leiomyoma has been reported in patients >35 years old. A total of 78% of ovarian leiomyoma cases are concomitantly observed with uterine leiomyoma and as hormone stimulation is a cause of uterine leiomyoma, it was hypothesized that hormone stimulation may also be a cause of ovarian leiomyoma (2). It is estimated that ~3% of gravid females present with an adnexal mass on ultrasound according to data obtained from 6636 pregnant females from the Department of Obstetrics and Gynecology in Italy between January 1996 and December 1999 (3). Surgery must be considered when the patient develops symptoms, such as abdominal pain or an ovarian cyst, or if the ovarian cyst is >6 cm in diameter, as this presents a risk to the continuing pregnancy (4). Few adnexal masses during pregnancy exhibit rapid growth with the progression of pregnancy. The current study presents the case of a 28-year-old female with an ovarian mass, which showed rapid growth during early pregnancy. The mass was excised by laparoscopy during the 12th week of gestation, without complications, when the mass had reached ~18×16×10 cm. The final postoperative histopathological examination confirmed primary ovarian leiomyoma. To the best of our knowledge, the management of large primary ovarian leiomyoma by laparoscopy during pregnancy has not been reported to date. Written informed consent was obtained from the patient.

## Case report

### Patient presentation

In October 2012, a 28-year-old female was referred to the local hospital due to two missed menstrual periods one month previously. Abdominal ultrasonography revealed a single normal fetus in the uterus and a right adnexal hypoechoic solid mass of 4.1×3.2 cm in size. The mass had been identified three years previously with a diameter of 2.5 cm during a routine gynecological check up and was followed-up by an ultrasound scan every six months for three years, which showed that the size of mass had remained stable. No further measurements were recorded due to the stability of the mass for a long period of time, without related symptoms. However, 16 days following the initial visit, the patient presented to the local hospital once again with a complaint of acute right lower abdominal pain following activity. An emergency ultrasound examination revealed that the adnexal mass had increased to 7×6.5 cm in size, without characteristics of torsion or rupture. The pain was significantly alleviated 1 h later without any therapy. Due to the increasing size of mass and the presence of pain, the patient was referred to Zhejiang Provincial People’s Hospital (Hangzhou, China) for surgical intervention.

During the following days, mild intermittent abdominal pain was experienced, without fever, vomiting, vaginal bleeding or uterine contractions. Approximately one week later the patient was admitted to Zhejiang Provincial People’s Hospital (Hangzhou, China) with slight abdominal pain on the right side. Physical examination revealed a tender mass of ~11×10 cm in size, located in the right upper abdominal quadrant region. The mass was palpable and movable, with clear edges. During the pelvic examination, the cervix was normal and no amniotic fluid or bloody discharge was observed. The uterus was movable with no tenderness, and was of the size corresponding to the gestational age.

**Figure 1:**
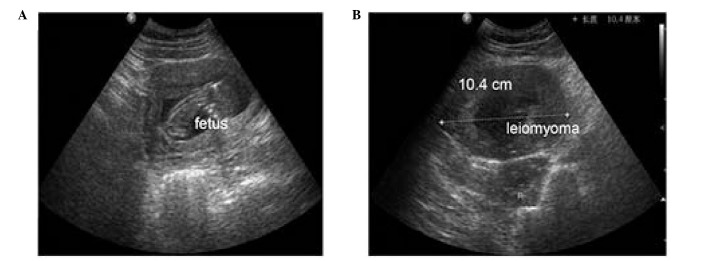
B-mode ultrasound showing (A) the fetus and (B) a right, hypoechoic adnexal mass.

Ultrasound revealed a single live fetus and a partial cystic adnexal mass of 10.4×10×6.6 cm in size (Fig. 1).

### Treatment

**Figure 2:**
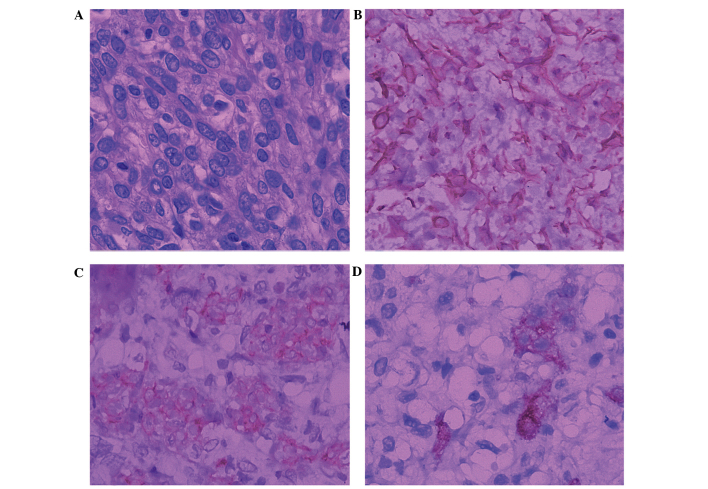
Hematoxylin and eosin (A) and immunohistochemical (B–D) staining (magnification ×400). (A) Typical fascicles of bland smooth muscle cells, (B) positive immunostaining for vimentin, (C) cells with granular inhibin staining and (D) positive staining for α-smooth muscle actin.

Surgery was scheduled to remove the mass one week later. The patient underwent laparoscopic ovarian tumorectomy under endotracheal intubation and general anesthesia. During the surgery, basic vital signs, including oxygen saturation and the end-tidal carbon dioxide level of the patient, were monitored continuously by the anesthetist. The laparoscope revealed an enlarged uterus of the size corresponding to that expected for a three-month pregnant uterus, normal bilateral fallopian tubes and a normal left ovary. A mass arising from the right ovary was ~18×16×10 cm in size and grossly appeared solid, with smooth/shiny surfaces and marked superficial vascularity. No adhesion or infiltration was observed in the pelvis. The ovary was preserved and the mass was excised. Following the completion of tumor enucleation, the ovarian defect was sutured with 2–0 absorbable VICRYL suture (Johnson & Johnson Medical Ltd., New Brunswick, NJ, USA) in a continuous manner, while bipolar electrocoagulation was used to secure hemostasis. The samples were removed using the bag-retrieval technique, where a 35×25 cm bag was used to remove the mass through the extended left trocar incision, and were subsequently sent to the Department of Pathology (Zhejiang Provincial People’s Hospital) for histological testing. The examination of frozen sections reported a benign spindle cell tumor with focal ischemic infarction. Pathological examination of the paraffin section following surgery demonstrated that the mass was comprised of typical smooth muscle cells, which formed strands and bundles arranged in a whorled interlacing pattern. Microscopically, ischemic infarction focus of the mass was observed, while significant nuclear atypia or pleomorphism was absent. Immunohistochemical staining with antibodies against vimentin, inhibin, α-smooth muscle actin and the α-helical rod domain of desmin was performed to confirm the diagnosis of this rare tumor. The tumor cells stained positively for vimentin, inhibin and α-smooth muscle actin, but not for the α-helical rod domain of desmin, which aided in confirming the diagnosis of ovarian leiomyoma (Fig. 2).

### Follow up

Fetal surveillance monitoring via ultrasound prior to and following surgery indicated that the surgery had been successful. Prophylactic antibiotics and progesterone at a dose of 20 mg twice daily for four days were prescribed following surgery to prevent miscarriage. The surgery time, defined as the period between the skin incision and the closure of the skin, was 118 minutes and the estimated blood loss was 800 ml. The patient experienced an uneventful postoperative recovery. Routine follow-up after discharge was conducted every six months for three years and no complications were observed. A 3.4-kg healthy baby was delivered spontaneously at full term.

## Discussion

Due to the widespread use of routine ultrasound in maternity examinations, it has been reported that the incidence of adnexal masses in pregnant females is ~3%, of which 55% may spontaneously resolve completely or significantly decrease in size (5). It is difficult to determine the treatment of adnexal masses in pregnancy, as surgery may pose a risk to the mother and fetus (6), but conservative management may result in severe complications, including torsion or rupture of the ovarian masses. However, there is no controversy with surgical intervention for pregnant females with symptoms of acute abdominal pain or adnexal mass larger than 6 cm in diameter (4).

The first case of a primary ovarian fibroid was described in 1862 (7,8), <100 cases of this rare tumor have since been reported (7,8). Clinically, few patients present with symptoms, and the majority of tumors are identified incidentally and are only a few millimeters in diameter (2,7). However, various characteristics of these benign neoplasms have been reported, including a variety of clinical presentations, such as abdominal pain, a palpable mass, marginal elevation in tumor marker levels and the complication of Meigs syndrome (8,9,10), as well as a wide range of tumor sizes. Lim and Jeon (8) described a case of large bilateral ovarian leiomyoma in a 17-year-old female in 2004, where the left ovary measured 17×10×7 cm and the right ovary measured 14×11×9 cm at their greatest dimensions. Similarly, Safaei *et al* (11) reported the case of a 54-year-old postmenopausal multipara female with an ovarian leiomyoma measuring 16×13×10 cm in size, in 2011. Furthermore, a study investigating uterine leiomyoma revealed that 15–30% of leiomyomas tend to grow under the influence of estrogens during pregnancy (12). Wei *et al* (8) suggested that estrogens may be a significant factor in the development of these ovarian neoplasms. A number of ovarian leiomyomas in pregnant females have been noted. Zorlu *et al* (13) presented the case of an adnexal mass complicating pregnancy, which was diagnosed as ovarian leiomyoma following surgery. The patient underwent surgery following spontaneous delivery, as there were no prior complaints with regard to the mass. In the case reported by Hsiao *et al* (14), the patient received an emergency cesarean section and oophorectomy due to an ovarian leiomyoma. The authors also drew the conclusion that increased concentrations of the progesterone and estrogen hormones during pregnancy may have been a factor in stimulating the growth of these tumors. Kohno *et al* (15) reported the case of a 32-year-old female who had a large pelvic mass, with rapid growth noted at the 16th week of gestation. An adnexal tumorectomy was performed on the patient by laparotomy at the 20th week of gestation, and the patient gave birth to a female infant at the 40th week of gestation. However, no recent studies have discussed the excision of an ovarian leiomyoma by laparoscopy during pregnancy. The case presented in the current study is of interest due to the rarity of this disease and the successful laparoscopic approach to remove the enlarged mass of the ovary during pregnancy.

We hypothesize that surgery is not to be considered for adnexal tumors of <3 cm in diameter during pregnancy. In the case presented in the current study, an adnexal tumor was observed in the patient, which had remained at ~2.5 cm in diameter for three years prior to gestation. However, the patient presented with symptoms of abdominal pain and rapid tumor growth during pregnancy, which suggested that surgical intervention prior to the birth should be considered for solid tumors identified before or during gestation, even for those <3 cm in diameter, in order to avoid complications during pregnancy.

Numerous studies have reported that a laparoscopic approach to the removal of ovarian masses provides benefits, including an improved view of the surgical site, reduced blood loss, fewer adhesions, decreased postoperative pain, shorter hospitalization and smaller scars, compared with transabdominal surgery. These advantages are particularly significant for gravid females. A laparoscopic approach for the management of benign ovarian tumors in pregnant females has become the preferred approach by surgeons, due to its efficacy and safety (16). Potential concerns with regard to laparoscopic surgery in pregnancy include a limited surgical field, trauma to the gravid uterus due to trocar penetration, and the negative effect on the fetus of CO_2_ gas pneumoperitoneum (17). The patient discussed in the current study was in the early second trimester of pregnancy, at which stage the uterus may not be of as much concern when placing the laparoscopic ports. Additionally, the correlation between CO_2_ abdominal insufflation and increased intraoperative complications during pregnancy has not been investigated to date. This case supports the previously observed safety and feasibility of laparoscopic approach in pregnancy.

The predominant challenges of laparoscopic tumorectomy of solid large ovary tumors during pregnancy are enucleation of the tumor and removal of the mass from the abdominal cavity, particularly when the mass exists in marked vascularity. During the surgical enucleation of large tumors with marked vascularity, bipolar and monopolar cautery, as well as ultrasonic scalpel devices were used to control bleeding, all of which were deemed entirely profitless exercises for the patient in the present case. Therefore, in this case, the tumor was enucleated and the residual ovary tissue was sutured rapidly for hemostasis, based on a skillful laparoscopic technique. However, ~800 ml of operative blood loss was observed. Temporary ligation of the ovarian pedicle, the proper ligament and the suspensory ligament may be promising in reducing operative blood loss; however, this approach was considered in this surgery. An additional challenge is the integral extraction of the solid tumor. Although the morcellation technique is prevalent in laparoscopic surgery for solid tumors, an endobag remains a necessity when removing the tumor, in order to minimize the probability of the diffusion and implantation of the tumor cells. During the surgery in the current case, the entire sample was enveloped in a 30×35-cm medical endobag, and removed via the trocar incision at the left anterior axillary line, which was extended to 3 cm following the initial scalpel incision.

In conclusion, to the best of our knowledge, no studies have investigated the laparoscopic management of primary ovarian leiomyomas during pregnancy. The case presented in the current study demonstrates that symptomatic ovarian leiomyoma may be successfully managed without complications by laparoscopic surgery, even when large in size and during pregnancy. By contrast, when considering solid progestational tumors, operative therapy prior to gestation is advised, even for tumors <3 cm in diameter, due to the probability of rapid growth of the tumor during pregnancy.
